# Developmental trends in young children’s device-measured physical activity and sedentary behaviour

**DOI:** 10.1186/s12966-024-01645-z

**Published:** 2024-09-02

**Authors:** Hayley E. Christian, Emma K. Adams, Hannah L. Moore, Andrea Nathan, Kevin Murray, Jasper Schipperijn, Stewart G. Trost

**Affiliations:** 1 Telethon Kids Institute, The University of Western Australia, 35 Stirling Highway, Perth, WA 6009 Australia; 2 School of Population and Global Health, The University of Western Australia, 35 Stirling Highway, Perth, WA 6009 Australia; 3 Department of Sports Science and Clinical Biomechanics, University of Southern Denmark, Campusvej 55, Odense, 5230 Denmark; 4 School of Human Movement and Nutrition Sciences, University of Queensland, Brisbane, QLD 4072 Australia

**Keywords:** Physical activity, Sedentary, Energetic play, Preschool, Children, Cohort, Longitudinal, Developmental, Guidelines, Machine learning

## Abstract

**Background:**

Knowledge of developmental trends in meeting age-specific 24-hour movement behaviour guidelines is lacking. This study describes developmental trends in device-measured physical activity and sedentary time over a three-year period among Western Australian children aged two to seven years, including differences between boys and girls. The proportion of children meeting age-specific physical activity guidelines before and after they transition to full-time school was also examined.

**Methods:**

Data from waves 1 and 2 of the Play Spaces and Environments for Children’s Physical Activity (PLAYCE) cohort study were used (analysis *n* = 1217). Physical activity and sedentary time were measured by accelerometry at ages two to five (preschool, wave 1) and ages five to seven (commenced full-time school, wave 2). Accelerometer data were processed using a validated machine-learning physical activity classification model. Daily time spent in sedentary behaviour, energetic play (moderate-to-vigorous physical activity (MVPA)), total physical activity, and meeting physical activity guidelines were analysed using linear and generalised linear mixed-effects models with age by sex interaction terms.

**Results:**

All movement behaviours changed significantly with increasing age, and trends were similar in boys and girls. Total daily physical activity increased from age two to five then declined to age seven. Mean daily total physical activity exceeded 180 min/day from ages two to five. Daily energetic play increased significantly from age two to seven, however, was below 60 min/day at all ages except for seven-year-old boys. Daily sedentary time decreased to age five then increased to age seven but remained lower than at age two. All two-year-olds met their age-specific physical activity guideline, decreasing to 5% of girls and 6% of boys at age four. At age seven, 46% of boys and 35% of girls met their age-specific physical activity guideline.

**Conclusions:**

Young children’s energetic play and total physical activity increased with age, but few children aged three to seven met the energetic play (MVPA) guideline. Interventions should focus on increasing children’s energetic play in early childhood. Clearer guidance and strategies are needed to support young children as they change developmentally and as they transition from one age-specific movement guideline to the next.

**Supplementary Information:**

The online version contains supplementary material available at 10.1186/s12966-024-01645-z.

## Background

Physical activity benefits children’s physical and mental health by supporting their motor skill development, musculoskeletal, cardiometabolic and psychological health, and preventing overweight and obesity [1–4]. Being physically active in childhood is associated with higher physical activity levels throughout the life course, where it has demonstrable effects on the prevalence of chronic diseases and mean life expectancy [5, 6].

Recently, international and country-specific physical activity guidelines for young children have been revised to encompass a full day’s movement behaviours including physical activity, sedentary behaviour, and sleep [7–11]. In Australia, the ‘24-Hour Movement Guidelines for the Early Years’ recommend children aged one to five years achieve at least 180 min of total physical activity each day, and, for preschool children aged three to five years, this should consist of at least 60 min of energetic play (moderate-to-vigorous intensity physical activity) [11, 12]. Once children transition to full-time school, the Australian ‘24-Hour Movement Guidelines for the Children and Young People’ recommend children aged 5–17 achieve at least 60 min of moderate-to-vigorous intensity physical activity each day [13]. While both movement behaviour guidelines for the ‘Early Years’ and ‘Children and Young People’ recommend limiting sedentary time, this is described specifically through age-specific screen time recommendations [12, 13].

Available evidence indicates that a large proportion of children do not meet physical activity recommendations [14–16]. There is limited device-based, longitudinal evidence on young children’s physical activity and how this varies by age, sex, physical activity intensity, and at key transitions such as moving to full-time school [17, 18]. The transition from preschool to full-time school is a notable shift in children’s physical and social environments that could have a marked influence on their movement behaviours [19]. Studies spanning this transition period have found children’s physical activity levels are generally higher during the preschool compared with full-time school period [18, 20]. However, some studies report physical activity levels are highest around three years of age [21] while others report five to six years [21–25], though not all studies include the transition to school period. It is unclear how the prevalence of meeting different movement behaviour guidelines changes from the preschool years to full time school attendance, particularly as they transition developmentally, and the amount and intensity of recommended movement behaviours changes from one guideline to the next. Further research is needed to determine how young children’s physical activity behaviours change over time and what impact this has on meeting age-specific movement behaviour guidelines.

A further challenge in determining the extent to which children meet physical activity guidelines is the variation in methods used to process device-measured movement data. In a systematic review of longitudinal studies using device-based measures of physical activity among children aged 2–18 years, six different cut-points were used to classify moderate-to-vigorous physical activity, the most common being the Evenson cut points for the ActiGraph accelerometer (43% of studies) [18]. However, recent research in young children demonstrates cut point methods consistently overestimate time in sedentary behaviour, underestimate time in light intensity physical activity, and overestimate time in moderate-to-vigorous intensity physical activity [26]. This misclassification of physical activity intensity has important implications for whether children meet or do not meet physical activity guidelines.

Recent updates to national [11] and international [8, 27] movement behaviour guidelines have called for more rigorous and consistent accelerometer data processing methods to inform future guideline development [27]. Machine learning classification methods can overcome the limitations of cut point methods. These methods provide more accurate assessments of physical activity intensity in young children when compared with traditional cut point methods and they can predict both physical activity type and intensity [26, 28]. However, to date, no longitudinal studies have used machine learning classification models to examine changes in children’s physical activity from early to middle childhood. As such, the small body of longitudinal evidence on children’s movement behaviours is limited by the reliance on cut-point based accelerometer data processing methods.

The aim of this study was to describe developmental trends in device-measured physical activity and sedentary time over a three-year period among Western Australian children aged two to seven years, including differences between boys and girls. The proportion of children meeting age-specific physical activity guidelines before and after they transitioning to full-time school was also examined.

## Methods

### Study design

This study uses data from the Play Spaces and Environments for Children’s Physical Activity (PLAYCE) cohort study. PLAYCE commenced as a cross-sectional observational study, collecting data from 1918 Western Australian children aged two to five during the period 2015 to 2018 (referred to as wave 1). Following this, families were invited to participate in the PLAYCE cohort study, which followed up 641 of the children from the original cross-sectional study as they transitioned to full-time school (wave 2, children aged five to eight) during the period 2018 to 2021. Details of the original study protocol have been published previously [29]. A STROBE checklist for the present study is in Additional File 1.

### Participants and setting

Children were recruited for wave 1 through their early childhood education and care (ECEC) service. Services were randomly selected to be invited to participate from a list of eligible services, stratified by the number of approved places (the maximum number of enrolled children) and service postcode-level socio-economic status [30]. After ECEC services provided consent to participate, all parents of children aged two to five attending the service were invited to participate. Children were ineligible if they had a recognised disability that would affect their participation in physical activity or if they were attending full-time school.

**Fig. 1:**
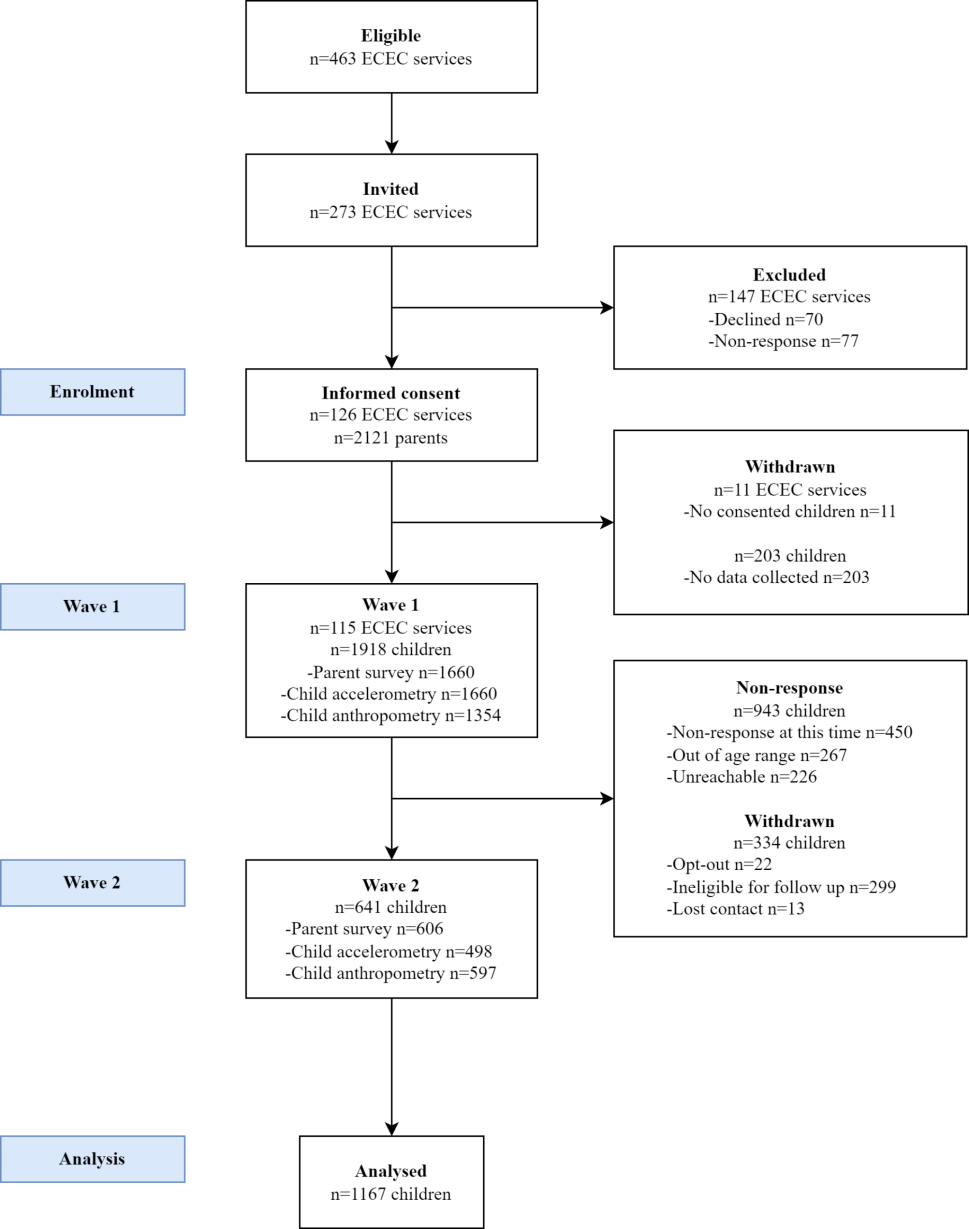
Flow diagram of PLAYCE study participation

Parents of eligible-aged children who participated in wave 1 were invited to participate in the PLAYCE cohort study. Parents were followed-up via email/SMS/telephone to confirm their willingness to participate. Children were ineligible at wave 2 if they had not provided data at wave 1, had not yet transitioned to full-time school, or were more than 8 years old. On average, children were followed up for wave 2 data collection three years after wave 1 participation. In Western Australia, children start full-time school the year they turn five years and six months old (i.e., they are aged between 4.5 and 5.5 years old when they commence school). The PLAYCE cohort participation flow chart is provided in Fig. 1.

### Sample size

The PLAYCE study aimed to recruit 120 ECEC services and 2,400 children [29] and wave 2 aimed to retain 70-to-80% of the original sample. Despite the wave 2 sample having greater attrition than expected, power calculations determined the study achieved 80% power to detect a 5-minute change in daily minutes of total physical activity between the two waves (effect size of 0.12), using an alpha level of 0.05 and a standard deviation of 42 min/day. The current analyses included 1,167 children aged 1.8 to 7.8 years from the PLAYCE cohort study who had valid accelerometer data for either wave 1 (*n* = 1,070) or wave 2 (*n* = 425) and all covariates. As no children were eight years old at wave 2, the study age range is referred to as two to seven years.

### Measures

#### Device-measured physical activity and sedentary time

Physical activity and sedentary time were measured using the ActiGraph GT3X + accelerometer (ActiGraph Corporation, Pensacola, FL USA). Children wore the devices on the right hip for seven consecutive days, excluding water activities. Since 24-hour wear was only incorporated from wave 2, sleep data were excluded from these analyses. Raw accelerometer data (30 Hz) were processed using a validated machine learned random forest physical activity classification model developed for young children [28].

The model was trained on fully annotated accelerometer data collected on 31 preschool-aged children who wore an ActiGraph GT3X + accelerometer on the hip and non-dominant wrist while completing a 20-minute free-living active play session. The location of the play session was chosen by the parent or caregiver (family home, park, local green space) and children were free to engage in any activity they desired. Each free play session was video recorded with a hand-held camera for subsequent direct observation coding using the Observer XT 14 software. A two-step coding scheme was implemented in which each child’s movement behaviour was first coded for physical activity intensity using the Children’s Activity Rating Scale (CARS) [31] and then activity type based on a list of 23 developmentally appropriate physical activities for preschool-aged children. The Observer XT software generated an event log comprising the start and end of each movement behaviour and a corresponding code for activity class based on the combination of physical activity type and intensity. Time stamps from the activity log was then used to calculate event duration and assign ground truth activity class labels to the corresponding time segment of the accelerometer data. Five broad activity classes served as prediction targets – sedentary (SED: sitting and lying down), light-intensity activity and games (L_ACT_G: e.g., slow walking or “pottering about”, standing, standing arts and crafts), moderate-to-vigorous intensity activities and games (MV_ACT_G: e.g., active games with balls, riding scooters/tricycles), walking (WALK) and running (RUN).

For model training and testing, fully annotated accelerometer data were segmented into 15-second non-overlapping windows or epochs. This provided over 2500 data instances for analysis, which was sufficient to train and test the classification model. Within each window, the tri-axial accelerometer signal was transformed into a single dimension vector magnitude and 25 time and frequency domain features were extracted and served as inputs to the random forest classification algorithm (500 trees). In leave-one-out cross-validation, recognition accuracy was as follows: SED (85.3%), L_ACT_G (92.3%), MV_ACT_G (72.0%), WALK (80.2%), and RUN (85.1%). Across all activity classes, the average F-Score (harmonic mean of precision and recall)) was 86%.

For the current study, predictions of activity class were mapped to traditional physical activity intensity categories as follows: SED windows were considered sedentary; L_ACT_G windows were considered light-intensity physical activity (LPA); while energetic play (MVPA) was calculated as the sum of MV_ACT_G, WALK, and RUN. Total physical activity was calculated by summing LPA and MVPA. In a follow-up study in which the random forest model was tested in a hold-out sample of 10 free-living preschoolers, the random forest model exhibited significantly higher agreement with directly observed physical activity intensity than previously published cut-points for preschool-aged children. Moreover, it was the only accelerometer data processing method to exhibit statistical evidence of equivalence with directly observed time in sedentary activity, light-intensity physical activity, and moderate-to-vigorous physical activity [26].

Non-wear periods were identified by summing the time periods in which the standard deviation of the accelerometer signal vector magnitude was < 13 mg for > = 30 consecutive minutes [32]. Children were included in the analysis if they had 4 or more valid monitoring days with at least 1 valid weekend day. Days were considered valid if wear time was ≥ 480 min per day. Children were defined as meeting physical activity guidelines based on the Australian 24-hr Movement Guidelines for the Early Years [12], and Australian 24-hr Movement Guidelines for Children and Young People [13]: total physical activity of ≥ 180 min/day for two-year-olds, total physical activity of ≥ 180 min/day including ≥ 60 min of energetic play for three- to five-year-olds (wave 1); or ≥ 60 min/day of energetic play (MVPA) for school children (wave 2).

#### Demographics

Parents completed a survey at each wave providing socio-demographic information on their work status, dwelling type, yard size, and the study child’s date of birth and sex.

### Statistical methods

Descriptive analysis was undertaken to examine the characteristics of participating children. Changes in children’s sedentary time and physical activity behaviours as a function of age were investigated using linear (for continuous data) or generalised linear (for meeting the physical activity guideline) mixed effects regression models (LMM and GLMM). Models included restricted cubic splines with four knots (consistent with recommendations by Harrell [33]) to account for a non-linear relationship between age and the dependent variables. All models included fixed effects for age, sex, and age-by-sex interaction, and random intercept effects to account for repeated measures on individuals. Models were also adjusted for family socio-demographics (wave 1 maternal work status, wave 1 dwelling type, wave 1 yard size), device wear time, and season of data collection. In addition, since wave 2 data spanned pre- and during- the COVID-19 pandemic, a fixed effect was included whereby accelerometer data captured on or after the date Western Australia entered a State of Emergency (March 15, 2020) was coded as during COVID-19. The final analysis sample was 1,167 children who had at least one wave of accelerometer data and all covariates. Data were analysed in R version 4.2.2 using the lme4 [34] and rms [33] packages. To aid interpretation of sedentary time and physical activity behaviour trajectories (referred to together as ‘movement behaviours’), marginal means for each movement behaviour were estimated and presented for ages two to seven using the ggeffects R package [35]. The proportion of children meeting the relevant physical activity guideline are not directly comparable between age two and age three due to the changing energetic play recommendation (from some energetic play to at least 60 min of energetic play/day); thus estimated proportions are presented in the figure only. Since there were no a priori hypotheses about differences in movement behaviours between specific ages (e.g., 4 vs. 3), these were not formally tested. Unadjusted estimates are presented in Additional File 2.

As the wave 2 sample was substantially smaller than the wave 1 sample, analyses were repeated including only children who had valid data at both waves (*n* = 326) to examine whether findings were related to the changing sample. These sensitivity analyses are presented in Additional File 3; there were no substantial differences in results compared to the main analyses.

## Results

### Participants

Approximately half of children were boys (51.5%, Table 1). Most children lived in a single residential dwelling (85.4%), had a medium or large sized yard (79.5%), and had a mother working full-time or part-time (81.1%) at wave 1. For both waves, accelerometer data were collected across all seasons. Around half of wave 2 accelerometer data were collected during the COVID-19 pandemic (46.1%). Characteristics of the analysis sample were similar to the full sample of the PLAYCE cohort.

**Table 1:** Characteristics of the PLAYCE cohort sample data

	All children	Analysis sample
	*n* = 1918	*n* = 1167
Wave 1 age (median years (IQR))	3.3 (1.2)	3.3 (1.1)
	*n* (%)	*n* (%)
Child sex		
Male	1011 (52.7)	601 (51.5)
Female	906 (47.3)	566 (48.5)
Mother’s work status		
Not in paid employment	311 (19.0)	221 (18.9)
Working full-time or part-time	1330 (81.0)	946 (81.1)
Dwelling type		
Standalone house	1333 (84.4)	997 (85.4)
Duplex/townhouse/flat/other	247 (15.6)	170 (14.6)
Yard size		
No or small yard	311 (19.8)	239 (20.5)
Medium or large yard	1260 (80.2)	928 (79.5)
Wave 1 valid accelerometer data^1^		
No	751 (39.2)	97 (8.3)
Yes	1167 (60.8)	1070 (91.7)
Wave 1 season		
Summer	127 (10.9)	113 (10.6)
Autumn	318 (27.2)	288 (26.9)
Winter	307 (26.3)	275 (25.7)
Spring	415 (35.6)	394 (36.8)
Wave 2 valid accelerometer data		
No	1474 (76.9)	742 (63.8)
Yes	444 (23.1)	425 (36.4)
Wave 2 season^1^		
Summer	71 (16.0)	68 (16.0)
Autumn	199 (44.8)	191 (44.9)
Winter	108 (24.3)	105 (24.7)
Spring	66 (18.9)	61 (14.4)
Wave 2 data collection during COVID-19^2^		
No	237 (53.4)	229 (53.9)
Yes	207 (46.6)	196 (46.1)
Wave 1 accelerometer wear time (mean mins/day (SD))	665.0 (64.3)	665.4 (63.9)
Wave 2 accelerometer wear time (mean mins/day (SD))	884.8 (154.4)	886.6 (154.8)

Table notes. Categories may not sum to denominator due to missing data. The analysis sample was restricted to children who had valid age and sex data, at least one wave of valid accelerometer data, and all covariates

^1^ Based on end date of accelerometer wear. Denominator is children with valid accelerometer data at relevant wave

^2^ Based on end date of accelerometer wear being on or after the date Western Australia entered a State of Emergency (15/03/2020). Denominator is children with valid accelerometer data at wave 2

### Daily movement behaviour

**Fig. 2:**
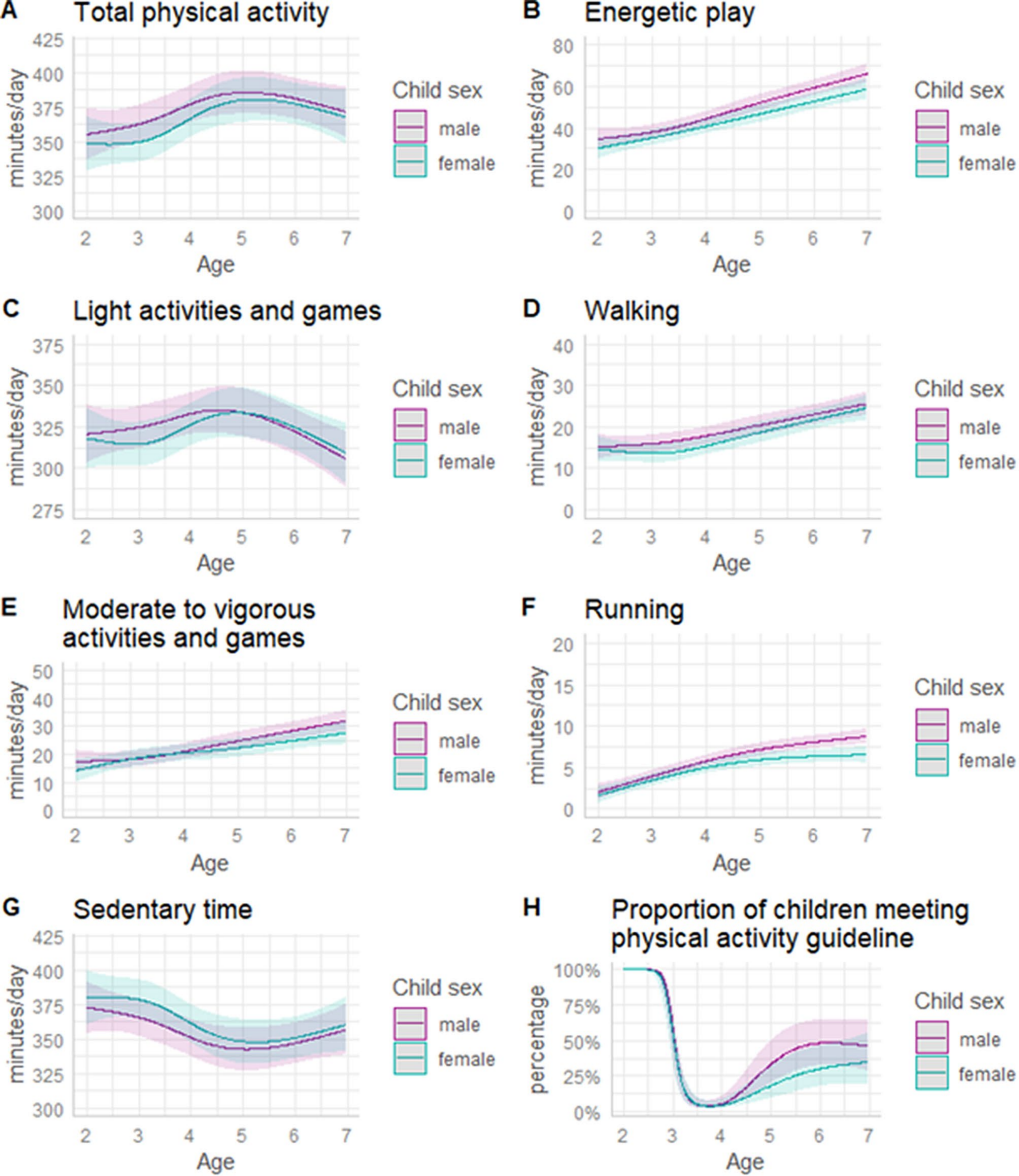
Estimated responses and proportions of children meeting age-specific energetic play guidelines in boys and girls aged 2 to 7 years. Figure notes. Graphs show means (or proportion for H) 95% confidence intervals estimated from LMM/GLMM adjusted for maternal work status, dwelling type, yard size, device wear time, season, data collection during COVID-19. Energetic play is the sum of walking, running, and moderate-vigorous activities and games. Total physical activity is the sum of energetic play and light intensity activities and games

Mean daily minutes of sedentary time and physical activity by intensity and type from ages two to seven are shown in Fig. 2; estimated marginal means for specific ages are presented in Table 2. Overall, movement behaviours differed significantly with increasing age (age main effects *p* < 0.05), with no evidence of any age by sex interactions.

Total daily physical activity changed non-linearly with increasing age (Fig. 2A); it increased from age two to five, then declined to age seven but remained higher than at age two. Daily energetic play (consisting of walking, running, and moderate-to-vigorous activities and games) increased significantly from age two to seven (Fig. 2B). Walking (Fig. 2D), running (Fig. 2F), and moderate-to-vigorous activities and games (Fig. 2E) all increased with increasing age. However, changes in daily minutes of running were significantly different between boys and girls (interaction *p* = 0.001); girls’ daily running plateaued after age five while boys’ daily running continued to increase with age. Time spent participating in light intensity activities and games (Fig. 2C) increased up to age five but thereafter declined to age seven; overall, time spent in light intensity activities and games was low at age seven than at age two. Finally, daily sedentary time (Fig. 2G) decreased from age two to five in boys and age three to five in girls, but subsequently increased slightly to age seven; overall, sedentary time was lower at age seven than at age two.

### Meeting physical activity guidelines

The proportion of children meeting age-specific physical activity guidelines varied significantly by age (Fig. 2H). At age two, all children met the 180 min/day of total physical activity guideline. All children aged three to five obtained at least 180 min/day of total physical activity. However, with the inclusion of the 60 min/day of energetic play requirement at age three, only 5–6% of girls and boys met the guideline. From age four the proportion meeting 180 min of total physical activity/day inclusive of 60 min/day of energetic play increased. By age seven half of boys (46.2%) and one-third of girls (35.3%) met the 60 min of energetic play/day guideline.

**Table 2:** Estimated marginal mean (95% CI) daily minutes of physical activity and sedentary time by age and sex

Age (years)	2	3	4	5	6	7
**Boys**						
Sedentary	372.8 (354.0, 391.6)	365.4 (351.8, 379.1)	350.8 (337.4, 364.2)	342.5 (326.9, 358.1)	346.9 (332.1, 361.7)	356.8 (338.5, 375.2)
Light intensity activities and games	320.7 (303.2, 338.2)	324.8 (312.2, 337.5)	333.1 (320.6, 345.6)	333.6 (319.1, 348.1)	322.0 (308.3, 335.8)	305.2 (288.1, 322.2)
Walking	15.2 (12.3, 18.1)	16.0 (13.9, 18.1)	17.9 (15.8, 20.0)	20.4 (18.0, 22.9)	23.1 (20.7, 25.4)	25.7 (22.8, 28.6)
Running	2.0 (1.1, 3.0)	3.9 (3.2, 4.6)	5.8 (5.1, 6.5)	7.2 (6.4, 7.9)	8.1 (7.3, 8.8)	8.8 (7.8, 9.7)
Moderate-vigorous activities and games	17.4 (13.4, 21.4)	18.2 (15.4, 21.1)	21.0 (18.2, 23.8)	24.8 (21.5, 28.1)	28.5 (25.4, 31.6)	32.0 (28.1, 35.9)
Energetic play	34.7 (29.9, 39.6)	38.0 (34.5, 41.5)	44.4 (41.0, 47.9)	52.2 (48.2, 56.2)	59.4 (55.6, 63.2)	66.4 (61.6, 71.1)
Total physical activity	355.4 (336.6, 374.3)	362.8 (349.2, 376.4)	377.5 (364.0, 390.9)	385.8 (370.2, 401.4)	381.4 (366.6, 396.1)	371.4 (353.0, 389.8)
**Girls**						
Sedentary	379.9 (360.2, 399.6)	378.4 (364.0, 392.8)	361.1 (347.4, 374.8)	348.0 (332.0, 364.0)	350.8 (335.8, 365.8)	360.6 (340.7, 380.4)
Light intensity activities and games	318.1 (299.8, 336.4)	314.7 (301.3, 328.1)	326.4 (313.7, 339.1)	333.5 (318.6, 348.4)	324.6 (310.7, 338.6)	308.9 (290.4, 327.3)
Walking	14.7 (11.6, 17.8)	13.6 (11.4, 15.9)	15.4 (13.3, 17.6)	18.7 (16.2, 21.2)	21.7 (19.4, 24.1)	24.7 (21.6, 27.8)
Running	1.6 (0.6, 2.6)	3.5 (2.7, 4.2)	5.0 (4.3, 5.7)	5.9 (5.1, 6.7)	6.3 (5.6, 7.1)	6.5 (5.6, 7.5)
Moderate-vigorous activities and games	14.3 (10.1, 18.4)	18.3 (15.3, 21.3)	20.5 (17.7, 23.4)	22.3 (19.0, 25.7)	24.9 (21.8, 28.0)	27.7 (23.5, 32.0)
Energetic play	30.3 (25.2, 35.4)	35.2 (31.5, 39.0)	40.8 (37.3, 44.4)	46.8 (42.7, 51.0)	52.9 (49.0, 56.7)	58.9 (53.8, 64.0)
Total physical activity	348.4 (328.7, 368.1)	349.9 (335.5, 364.3)	367.1 (353.5, 380.8)	380.2 (364.2, 396.2)	377.4 (362.5, 392.4)	367.7 (347.8, 387.6)

Table notes. Marginal means estimated from LMM adjusted for maternal work status, dwelling type, yard size, device wear time, season, and data collection during COVID-19

Energetic play is the sum of walking, running, and moderate-vigorous activities and games

Total physical activity is the sum of energetic play and light intensity activities and games

Main effects for age were significant (*p* < 0.05) for boys and girls for all measures. Main effects for sex were non-significant for all measures (*p* > 0.05). Age by sex interaction was significant only for running (*p* = 0.001), for all other measures age by sex interaction was non-significant *p* > 0.05

## Discussion

The PLAYCE cohort study used device-measured movement behaviours and machine learning classification methods to investigate changes in children’s physical activity and sedentary behaviours and adherence with Australian child-based 24-Hour Movement Guidelines (physical activity component) between the ages of two and seven years old. Our results demonstrated daily total physical activity minutes increased from age two to five and declined marginally thereafter, and daily minutes of energetic play increased from age two to seven. Daily sedentary time also showed age-related changes, though remained highest from age two to three. Overall, physical activity and sedentary time were similar for boys and girls across all ages.

The extent to which children met Australian age-specific movement behaviour guidelines was also examined. All two-year-olds met the recommended 180 min of total physical activity per day. For three- to five-year-olds the additional guideline requirement of 60 min/day of energetic play meant guideline adherence dropped to only 5–6% at age four. Adherence to 60 min/day of energetic play gradually increased with age and by seven about half of boys and one-third of girls did 60 min or more of energetic play per day.

Few studies have examined developmental changes in young children’s physical activity and sedentary behaviours using accelerometry. Of the few studies conducted, all used cut point accelerometer data processing methods which are limited due to the underestimation of time in light intensity physical activity and overestimation of time in moderate-to-vigorous intensity physical activity [26]. Despite the current study using more accurate machine learning physical activity classification methods [26, 28], our finding that daily total physical activity increased from age two to five years old and then declined is in support of findings from other large studies. For example, pooled data from the International Children’s Accelerometry Database (ICAD) [17] including 20, primarily cross-sectional studies from 10 countries showed total physical activity was highest at age five to six and thereafter declined through middle childhood and adolescence. In addition, recent longitudinal research from Norway [22] and Canada [36] also show similar patterns of total daily physical activity increasing to around five years and then declining.

However, our findings related to the developmental changes in young children’s light intensity physical activity and energetic play are in contrast to previous studies. We found light intensity physical activity increased to age five while other studies report young children’s light intensity physical activity declines with increasing age [17, 21, 22]. We also identified linear increases in daily time spent in energetic play from age two to seven, yet other research has suggested energetic play (MVPA) plateaued or declined after age five to six [17, 22, 37, 38] or did not change over early to middle childhood [21]. It is likely the different accelerometer processing methods used in the current study (machine learning classification models compared with cut points) may explain these contrasting results. Longer follow-up of children in the ongoing PLAYCE cohort study will help clarify trends in children’s physical activity through middle childhood.

Our findings showed all children aged two to five achieved at least 180 min of total physical activity per day. This is consistent with findings in other longitudinal research with preschool-aged children, using cut point accelerometer data processing methods [39–41]. However, only one-third of three to five years olds met the 180 min of total physical activity, when 60 min of energetic play per day is included in the guideline. This was driven by the proportion of three- to five-year-olds meeting/not meeting the 60 min of energetic play per day component of the guideline. The proportion of school-aged children achieving the recommended 60 min of energetic play per day (MVPA) was approximately 50% for six- to seven-year-old boys and about one third for six- to seven-year-old girls. Overall, most children aged three to seven years did not achieve sufficient amounts of daily energetic play according to Australian child-based 24-Hour Movement Guidelines. These findings are in contrast to other research which generally report higher proportions of preschool [14] and school-aged [1, 42] children meeting energetic play/MVPA guidelines, using cut point accelerometer data processing methods.

Time spent being sedentary was highest among children aged two to three years and lowest among children aged five years, the age at which Western Australian children commence fulltime school. While sedentary time increased marginally at ages six and seven, it remained below levels observed among two- to three-year-old children. This is possibly due to young children’s increasing ability to be physically active as they develop fundamental movement skills and engage in more structured physical activity (e.g., swimming, dance lessons). Importantly, the Australian 24-Hour Movement Guidelines for children operationalises sedentary behaviour sedentary screen time, which cannot be specifically measured by accelerometry. In addition, there are types of sedentary behaviour such as sitting and reading books that are beneficial for a child’s development. Subjective measures (e.g., parent report) suggest few children meet age-specific screen time guidelines [14, 16, 43]. Future research should consider the use of both accelerometry as well as parent-report measures to gain a better understanding of the developmental trends in young children’s sedentary behaviour.

The changing movement guidelines for young children create a complexity in investigating the extent to which children meet guidelines. For example, children aged two years old are recommended to a minimum of 180 min of total daily physical activity, while children aged three to five need at least 60 min of daily energetic play as part of their 180 min of total daily physical activity [12]. In the current study, variation in meeting the physical activity guideline for preschool aged children was only evident in the energetic play component; all preschool children achieved 180 min of total physical activity but only one-third achieved the recommended 60 min of energetic play per day. Based on our findings, a child turning three would need to increase their daily energetic play by up to twenty-five minutes per day to meet the 180 min of total physical activity including at least 60 min of energetic play per day. In addition, although energetic play increased as children got older, mean daily energetic play was below the 60-minute guideline for school-aged children except for among seven-year-old boys (mean 66.4 min/day). Future 24-Hour Movement Guideline development and intervention strategies should focus on promoting children’s energetic play in early childhood.

There was little difference in the developmental trends of boys’ and girls’ movement behaviours. However, school aged girls (six- to seven-year-olds) spent less time in running activities than boys, and fewer school-aged girls achieved the recommended 60 min of energetic play (MVPA) per day than boys. This is somewhat in contrast to previous research showing sex differences are apparent in children as young as two years old [20, 44]. The differences in findings may be in part explained by the different methods used to process accelerometer data.

We have demonstrated the use of machine learning classification models to process device-measured physical activity which can be applied consistently and longitudinally to more accurately understand trends in young children’s movement behaviours. This is in line with the World Health Organization’s recommendation to develop harmonised methods of measuring and processing device-based estimates of physical activity [27]. Adoption of machine learning classification methods to process device-based movement behaviour data will improve comparability between studies, provide more consistent results, and allow more accurate population monitoring of children’s movement behaviours over time, thereby better informing future intervention strategies. In addition, future studies should examine developmental trends in young children’s sleep as well as physical activity and sedentary behaviour and consider composition data analysis methods [45–47] to examine the combination of movement behaviours across the whole day, and how substitutions in movement behaviours are associated with different health and development outcomes.

### Strengths and limitations

A strength of this study was the use of device-measured physical activity data processed using a machine learning classification method that overcomes major limitations of using traditional cut point processing methods. Furthermore, the study included a large cohort of preschool children followed up as they transitioned to full time school (aged two to seven years old). Given the non-linear developmental pattern of young children’s movement behaviours, a strength of this study was its use of non-linear analysis methods.

A main limitation of this study was the drop out of participants at wave 2. While there were small demographic differences between children only participating at wave 1 compared to those who participated at both waves, sensitivity analyses did not reveal differences in physical activity trends compared to the main analysis. Further cohort studies using large representative samples followed up over multiple time points from early to middle childhood are needed to confirm these findings. As well, the random forest physical activity classification model was trained on free-living data collected in preschool-aged children. Thus, the extent to which the model generalises to children aged six and seven years has not been formally investigated. However, as this was a longitudinal analysis, it was important to retain the same accelerometer data processing method to ensure the results could be compared over time. It is important future research continues to develop and test machine learning classification models for children of wider age ranges. It is also important to acknowledge that the machine learning classification model was not without error. Under true free-living conditions, Ahmadi et al. observed that a small percentage of true sedentary windows were misclassified as light intensity activity and games if they were performed with significant upper body movement [48]. Conversely, periods of standing were occasionally misclassified as sedentary if upper body movements were minimal. Additionally, although recognition of moderate to vigorous activity and games was acceptable, a relatively small percentage of true moderate to vigorous activity and games windows were misclassified as light intensity activity and games. Finally, this study was not able to include sleep in investigating the developmental trends of young children’s movement behaviours. Future research should consider developmental trends in all three movement behaviours (physical activity, sedentary behaviour and sleep) as well as the use of specific measures of sedentary behaviour such as parent-reported sedentary leisure screen time.

## Conclusions

Results from this study highlight most children aged three to seven years are falling short of achieving the recommended 60 min per day of energetic play. As children got older, light intensity and total physical activity declined while energetic play increased but generally remained below recommended levels. These findings highlight the importance of selecting appropriate data processing methods when using device-based movement behaviour data as it can significantly impact the proportion of children meet or not meeting 24-Hour Movement Behaviour Guidelines. Furthermore, the methods used to process device-based movement data can also influence where and how to target intervention strategies to enable young children to engage in sufficient energetic play to support their health and development. To achieve Australian child-based 24-Hour Movement Behaviour Guidelines, increased intervention is needed to improve children’s energetic play (MVPA). Clearer guidance and strategies are also needed to support children as they transition from one age-specific guideline to the next.

### Electronic supplementary material

Below is the link to the electronic supplementary material.

Additional. Table 1. STROBE checklist for cohort studies.

Additional Table 2. Unadjusted mean daily sedentary time and physical activity estimates.

Additional Table 3. Sensitivity analyses: Marginal mean daily sedentary time and physical activity estimates.

## Data Availability

The datasets used during the current study are available through application to the PLAYCE cohort study.

## Abbreviations

COVID: 19 Coronavirus disease of 2019
ECEC: Early Childhood Education and Care
GLMM: Generalised linear mixed effects model
LMM: Linear mixed effects model
MVPA: Moderate-to-vigorous physical activity
PLAYCE: Play Spaces and Environments for Children’s Physical Activity
STROBE: Strengthening the reporting of observational studies in epidemiology
