# Supplementary material for: Developmental trends in young children’s device-measured physical activity and sedentary behaviour
Source: Int J Behav Nutr Phys Act. 2024 Sep 2;21:97. doi: 10.1186/s12966-024-01645-z (PMC11370073; doi:10.1186/s12966-024-01645-z)
Supplement: Supplementary file 2 — Additional Table 2. Unadjusted mean daily sedentary time and physical activity estimates. [file 12966_2024_1645_MOESM2_ESM.docx]

Additional Table 2. Unadjusted mean (95% CI) daily minutes of physical activity and sedentary time by age and sex.

| **Age (years)** | 2 | 3 | 4 | 5 | 6 | 7 |
| --- | --- | --- | --- | --- | --- | --- |
| **Boys** |  |  |  |  |  |  |
| Sedentary | 246.6 (233.9, 259.4) | 289.7 (280.4, 298.9) | 332.7 (325.2, 340.2) | 375.7 (367.1, 384.3) | 418.7 (407, 430.5) | 461.8 (446, 477.5) |
| Light intensity activities and games | 327.6 (320.5, 334.6) | 336.7 (331.5, 341.9) | 345.8 (341.6, 350.1) | 355.0 (350.1, 359.9) | 364.1 (357.5, 370.8) | 373.3 (364.5, 382.1) |
| Walking | 15.5 (14.4, 16.6) | 18.0 (17.2, 18.8) | 20.5 (19.8, 21.2) | 23.0 (22.2, 23.8) | 25.5 (24.5, 26.6) | 28 (26.7, 29.4) |
| Running | 2.7 (2.4, 3.1) | 4.2 (3.9, 4.4) | 5.6 (5.4, 5.8) | 7.0 (6.8, 7.3) | 8.5 (8.1, 8.8) | 9.9 (9.5, 10.3) |
| Moderate-vigorous activities and games | 14.1 (12.5, 15.8) | 17.3 (16.1, 18.5) | 20.5 (19.5, 21.5) | 23.7 (22.6, 24.8) | 26.9 (25.3, 28.4) | 30.0 (28, 32.1) |
| Energetic play | 32.3 (30.4, 34.3) | 39.5 (38.1, 40.9) | 46.6 (45.4, 47.8) | 53.7 (52.4, 55.1) | 60.9 (59.1, 62.6) | 68.0 (65.6, 70.4) |
| Total physical activity | 359.9 (352.3, 367.4) | 376.2 (370.6, 381.7) | 392.4 (387.9, 397.0) | 408.7 (403.5, 413.9) | 425.0 (418.0, 432.0) | 441.3 (431.9, 450.7) |
| % meeting physical activity guideline | 100.0 (100.0, 100.0) | 59.1 (49.1, 68.3) | 6.3 (4.2, 9.5) | 35.7 (27.6, 44.8) | 51.4 (44.7, 58.1) | 50.6 (40.5, 60.6) |
| **Girls** |  |  |  |  |  |  |
| Sedentary | 240.7 (227.2, 254.2) | 285.7 (276, 295.4) | 330.7 (322.9, 338.4) | 375.6 (366.8, 384.5) | 420.6 (408.4, 432.9) | 465.6 (449.1, 482.2) |
| Light intensity activities and games | 315.2 (307.7, 322.6) | 327.4 (321.9, 332.8) | 339.6 (335.2, 344) | 351.8 (346.8, 356.9) | 364.1 (357.2, 371) | 376.3 (367.1, 385.6) |
| Walking | 13.2 (12.0, 14.4) | 15.8 (15, 16.7) | 18.5 (17.8, 19.2) | 21.2 (20.4, 22) | 23.8 (22.7, 24.9) | 26.5 (25, 27.9) |
| Running | 2.6 (2.2, 3.0) | 3.6 (3.4, 3.9) | 4.7 (4.4, 4.9) | 5.7 (5.5, 6.0) | 6.8 (6.4, 7.1) | 7.8 (7.4, 8.3) |
| Moderate-vigorous activities and games | 14.6 (12.9, 16.4) | 16.7 (15.4, 17.9) | 18.7 (17.7, 19.7) | 20.8 (19.7, 21.9) | 22.9 (21.3, 24.4) | 24.9 (22.8, 27.1) |
| Energetic play | 30.4 (28.4, 32.4) | 36.2 (34.7, 37.7) | 42 (40.8, 43.2) | 47.8 (46.4, 49.1) | 53.5 (51.7, 55.4) | 59.3 (56.8, 61.8) |
| Total physical activity | 345.6 (337.6, 353.5) | 363.6 (357.8, 369.4) | 381.6 (376.9, 386.3) | 399.6 (394.2, 405) | 417.6 (410.3, 424.9) | 435.6 (425.8, 445.5) |
| % meeting physical activity guideline | 100.0 (100.0, 100.0) | 55.3 (44.5,65.7) | 4.8 (3.1, 7.4) | 19.7 (13.1, 28.5) | 32.8 (26.4, 39.9) | 38.8 (28.0, 50.9) |

Energetic play is the sum of walking, running, and moderate-vigorous activities and games.

Total physical activity is the sum of energetic play and light intensity activities and games.

Comparison of % meeting physical activity guideline are not directly comparable between age 2 and age 3 due to the changing requirement regarding energetic play.

Main effects for age were significant (p<0.001) for boys and girls for all measures. Main effects for sex were significant for light intensity activities and games only (p<0.05). Age by sex interaction was significant for running, energetic play, and moderate-vigorous activities and games (p<0.05).
