# Supplementary material for: Developmental trends in young children’s device-measured physical activity and sedentary behaviour
Source: Int J Behav Nutr Phys Act. 2024 Sep 2;21:97. doi: 10.1186/s12966-024-01645-z (PMC11370073; doi:10.1186/s12966-024-01645-z)
Supplement: Supplementary file 3 — Additional Table 3. Sensitivity analyses: Marginal mean daily sedentary time and physical activity estimates. [file 12966_2024_1645_MOESM3_ESM.docx]

Sensitivity analyses:

Analyses were repeated including only children who had data at both waves to examine whether findings were related to the changing sample. In total, 326 children with valid data and all covariates were entered into the model. There were no substantial differences in results compared to the main analyses, except daily minutes of light intensity activities and games did not change over time for boys or girls (age main effects p>0.05) and the change in energetic play was significantly different for boys and girls (interaction p<0.05). Additional Table 3 presents the marginal means for each behaviour.

Additional Table 3. Estimated marginal mean (95% CI) daily minutes of physical activity and sedentary time by age and sex.

| **Age (years)** | 2 | 3 | 4 | 5 | 6 | 7 |
| --- | --- | --- | --- | --- | --- | --- |
| **Boys** |  |  |  |  |  |  |
| Sedentary | 441.4 (405.8, 477.0) | 424.6 (398.6, 450.6) | 408.9 (382.1, 435.7) | 397.7 (370.7, 424.7) | 396.0 (368.0, 423.9) | 405.1 (373.9, 436.4) |
| Light intensity activities and games | 302.5 (268.8, 336.2) | 319.0 (294.4, 343.7) | 327.9 (302.5, 353.3) | 326.4 (300.8, 352.0) | 319.7 (293.2, 346.2) | 312.3 (282.7, 341.8) |
| Walking | 13.2 (8.0, 18.4) | 14.0 (10.2, 17.8) | 15.9 (12.0, 19.9) | 19.2 (15.3, 23.1) | 22.1 (18.1, 26.2) | 23.8 (19.3, 28.3) |
| Running | 1.3 (-0.4, 3.0) | 2.9 (1.6, 4.2) | 5.2 (3.8, 6.5) | 7.7 (6.3, 9.0) | 8.7 (7.3, 10.1) | 7.4 (5.8, 8.9) |
| Moderate-vigorous activities and games | 18.8 (12.2, 25.5) | 17.7 (13.0, 22.4) | 20.7 (15.8, 25.6) | 27.5 (22.6, 32.4) | 31.8 (26.6, 36.9) | 29.8 (24.0, 35.6) |
| Energetic play | 33.9 (25.8, 41.9) | 34.5 (28.7, 40.4) | 41.4 (35.4, 47.5) | 54.1 (48.0, 60.2) | 62.5 (56.2, 68.8) | 60.8 (53.7, 67.8) |
| Total physical activity | 336.7 (301.1, 372.3) | 353.5 (327.5, 379.5) | 369.2 (342.4, 396.0) | 380.4 (353.4, 407.4) | 382.1 (354.2, 410.1) | 373.0 (341.7, 404.2) |
| **Girls** |  |  |  |  |  |  |
| Sedentary | 440.6 (404.9, 476.4) | 433.0 (405.7, 460.3) | 420.9 (392.4, 449.3) | 407.3 (378.0, 436.7) | 404.7 (375.3, 434.0) | 419.0 (386.7, 451.3) |
| Light intensity activities and games | 307.6 (273.8, 341.5) | 311.5 (285.6, 337.4) | 317.8 (290.9, 344.8) | 323.9 (296.1, 351.8) | 320.7 (292.8, 348.5) | 303.9 (273.3, 334.5) |
| Walking | 13.9 (8.8, 19.1) | 12.6 (8.6, 16.5) | 13.7 (9.5, 17.8) | 17.5 (13.2, 21.8) | 20.9 (16.6, 25.2) | 21.9 (17.2, 26.6) |
| Running | 1.0 (-0.8, 2.7) | 2.7 (1.3, 4.1) | 4.3 (2.9, 5.8) | 5.7 (4.2, 7.2) | 6.3 (4.8, 7.7) | 5.8 (4.2, 7.4) |
| Moderate-vigorous activities and games | 15.4 (8.8, 22.0) | 18.4 (13.4, 23.4) | 21.3 (16.1, 26.5) | 23.8 (18.5, 29.2) | 25.9 (20.5, 31.3) | 27.4 (21.5, 33.4) |
| Energetic play | 29.8 (21.7, 37.9) | 33.6 (27.4, 39.8) | 39.4 (33.0, 45.8) | 46.9 (40.3, 53.6) | 52.9 (46.2, 59.5) | 55.3 (48.0, 62.6) |
| Total physical activity | 337.5 (301.7, 373.2) | 345.1 (317.8, 372.4) | 357.2 (328.8, 385.7) | 370.8 (341.4, 400.1) | 373.4 (344.1, 402.8) | 359.1 (326.8, 391.4) |

Notes. Models include only those children who had valid wave 1 and wave 2accelerometer data. Means estimated from LMM adjusted for maternal work status, dwelling type, yard size, device wear time, season, and data collection during COVID-19.

Energetic play is the sum of walking, running, and moderate-vigorous activities and games.

Total physical activity is the sum of energetic play and light intensity activities and games.

Main effects for age were significant (p<0.05) for boys and girls for all measures except light intensity activities and games. Main effects for sex were non-significant for all measures (p>0.05). Age by sex interaction was significant for energetic play and running (p<0.05), for all other measures age by sex interaction was non-significant p>0.05.
